# Synthesis of diamond-like phase from graphite by ultrafast laser driven dynamical compression

**DOI:** 10.1038/srep11812

**Published:** 2015-07-07

**Authors:** Francisco C. B. Maia, Ricardo E. Samad, Jefferson Bettini, Raul O. Freitas, Nilson D. Vieira Junior, Narcizo M. Souza-Neto

**Affiliations:** 1 Laboratório Nacional de Luz Síncrotron (LNLS), Campinas, São Paulo 13083-970, Brazil; 2 Instituto de Pesquisas Energéticas e Nucleares (IPEN-CNEN/SP), São Paulo 05508-000, Brazil; 3 Laboratório Nacional de Nanotecnologia (LNNano), Campinas, São Paulo 13083-970, Brazil

## Abstract

Rapid variations of the environmental energy caused by ultrashort laser pulses have induced phase transitions in carbon allotropes, therefore bringing the promise of revealing new carbon phases. Here, by exposing polycrystalline graphite to 25 fs laser pulses at 4 J/cm^2^ fluence under standard air atmosphere, we demonstrated the synthesis of translucent micrometer-sized structures carrying diamond-like and onion-like carbon phases. Texturized domains of the diamond phase were also identified. Concerning different synthesized carbon forms, pulse superposition and singularities of the thermodynamical process, we pinpoint the synthesis mechanism by the laser-induced subsequent products energetically evolving to attain the diamond-like phase.

In recent years carbon allotropes, such as nanodiamonds, have shown promising new applications in many fields due to its physical, chemical and surface characteristics. Their high electron mobility, field electron emission and magnetic properties^1^ make them important players in carbon based electronics^2^,^3^. Their tribological and mechanical^1^,^4^,^5^ properties give rise to harder coatings^1^,^5^, which are biocompatible and can provide improved biological prosthesis joints^6^ with decreased wear. This biocompatibility allied to their biosensing, optical and nanoprobing functionalities^7^,^8^ provide drug delivery and cellular labeling capacity^7^. This wide range of novel applications has fostered the active search for new and more efficient synthesis and production methods of carbon allotropes.

In this pursuit, it has been shown that dynamical compression is a way to steer carbon allotropes, through rapid energy variations, to phase transitions. As examples, transition from graphite to diamond headed by shock compression experiments occurs through the fast martensitic mechanism^9^; in a setup designed for quenching shock-compressed samples at a fast cooling rate, the n-diamond phase^10^ has been created, and in regions of shock impacted meteorites nanodiamonds have been formed^11^. These observations brought to light the importance of carbon phase transformations in non-equilibrium states, spurring studies in a faster time scale using ultrashort pulses, which trigger shockwaves^12^,^13^ carrying extreme temperature and pressure in the matter. Experiments employing high peak power ultrashort laser pulses with durations around 100 femtoseconds irradiating Highly Oriented Pyrolytic Graphite (HOPG), usually under vacuum^14^,^15^,^16^ or in the graphite/liquid interface^17^, have originated sp^3^-bounded lattices on the graphite^14^,^18^ and provided preliminary evidences of diamond formation on the substrates^14^,^15^,^16^,^17^,^19^,^20^. Ultrashort laser shots raise promises for synthesizing known^21^ and still theoretically predicted^22^,^23^ sp^3^ carbon structures. Here, we have taken advantage of moderate energy ultrashort laser pulses to generate shockwaves and induce the formation of a diamond-like phase from the sp^2^ carbon polycrystalline graphite precursor.

In the present work, laser shots irradiating graphite under air dramatically modified the surface and created micrometer scale translucent structures where diamond-like crystallites were found coexisting with onion-like phases and quasi-amorphous nanometer sized graphite. Such materials were recovered after the laser irradiation process and analyzed by micro Raman spectroscopy, Scanning Electron Microscopy (SEM) and High Resolution Electron Microscopy (HREM). Considering the ultrafast optical excitation, ablation regime and the distinct laser-synthesized carbon structures, we propose an indirect mechanism in which subsequent laser-induced transformations lead to the synthesis of the diamond-like phase.

## Results and Discussion

**Figure 1:**
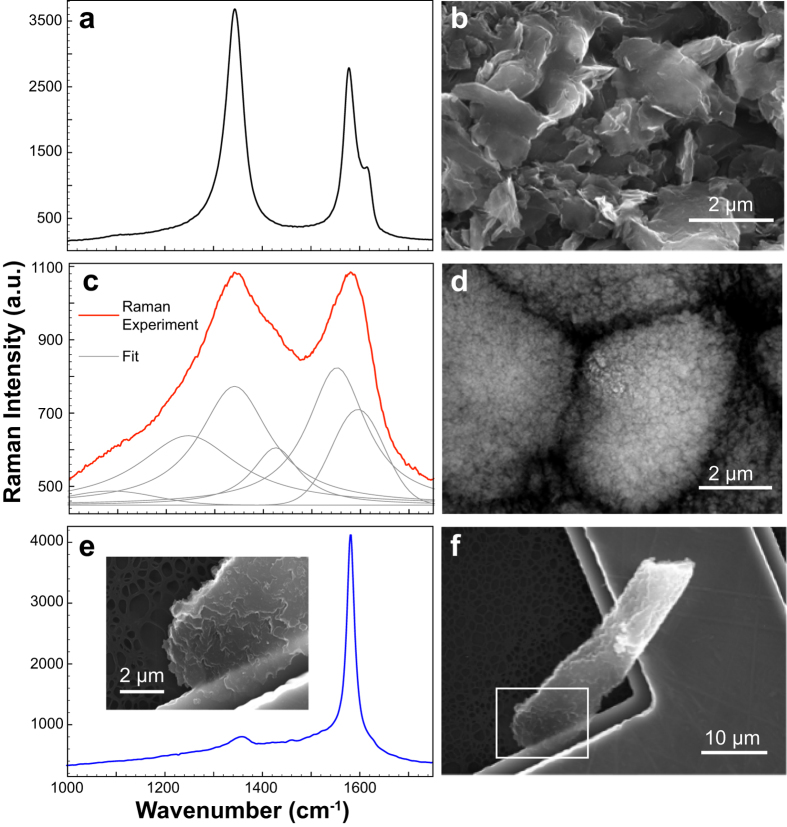
On the left are shown the Raman spectra and on the right the respective SEM images. (**a**) and (**b**) refer to the polycrystalline graphite (precursor). (**c**) and (**d**) correspond to the laser-modified surface, and in (**c**), the thicker red curve is the measured Raman spectrum and the gray ones are its component bands obtained from Voigt functions fittings. (**e**) and (**f**) present the measurements for the laser-created structure (the corresponding optical image is shown in the Figure S1 of the Supplementary Information, which also shows the Raman spectra for two other laser created particles). Clear morphological differences are noted between the precursor and the laser modified materials. The raw graphite contains disordered and buckled flakes (**b**). In (**d**) the material features micrometer sized globular formations composed by nanometer sized structures. Stacked layers resulting from shockwaves dynamic compressed carbon produce the structure shown in (**e**).

The Raman spectra and the SEM micrographs shown in Fig. 1 present the evolution of the induced modifications in the ultrashort laser pulses irradiated polycrystalline graphite sample through assessing the stages from the precursor (Fig. 1a,b), the intermediary stage found at the irradiated surface (Fig. 1c,d), to the final diamond-like structure (Fig. 1e,f).

In the Fig. 1a the pristine graphite exhibits its characteristic Raman modes at 1343 and 1615 cm^−1^ (related to structural defects bands D1 and D2, respectively), and at 1579 cm^−1^ originating from the stretching of the sp^2^-bounded carbon atoms forming the well-known hexagonal structure (G-band)^24^. After irradiation, notable changes were observed in the Raman spectrum of the laser modified surface, shown in Fig. 1c, with respect to the precursor (Fig. 1a). While the D1 band appears at 1340 cm^−1^, the displacement of the G band to 1595 cm^−1^ (Fig. 1c) confirms the laser-induced pressure, since similar shifts to higher wavenumbers were verified for HOPG upon room-temperature compression^25^. Additionally, Raman resonances, which do not manifest in the precursor (Fig. 1a), emerge at 1087, 1245, 1425 and 1552 cm^−1^. Analogous spectra have been reported for an experiment that quenched Carbon Black from high-pressure high-temperature environment^26^ (15 GPa and 1700 °C for 15 min) and also in shocked meteorites^27^. In the former work, the new vibrations were declared unexplained Raman modes, while in the latter they were attributed to a new carbon phase. Considering previous works^26^,^27^,^28^ and our experimental observations, we suggest that these vibrations are evidence of the creation of sp^3^ carbon phases by the ultrafast laser excitation, as theoretical models^29^ have predicted sp^3^ lattices with rather specific Raman resonances.

**Figure 2:**
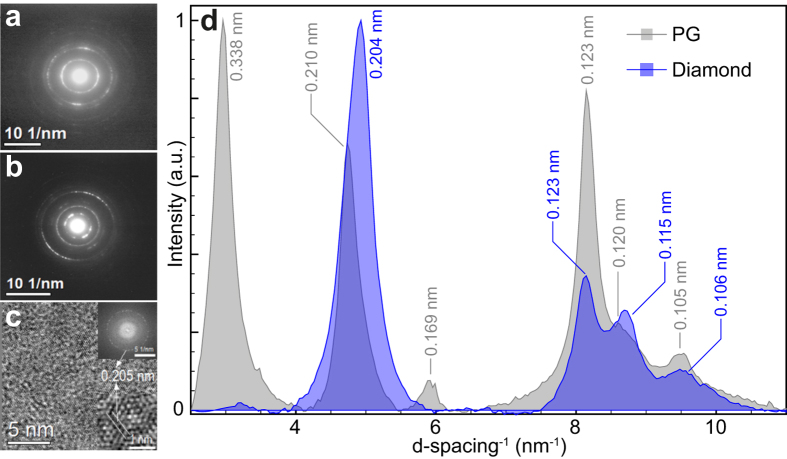
High Resolution Electron Diffraction analysis of the diamond-like phase. SADs of the (**a**) laser created structures, and (**b**) its graphite precursor; (**c**) HREM micrograph of the laser created structure with the characteristic 0.205 nm d-spacing of the diamond phase evidenced by the zoom of a small area shown in the bottom inset; the upper inset shows the Fourier Transform of the whole image; (**d**) exhibits the corresponding electron diffraction peaks as a function of the inverse of the d-spacing for the polycrystalline graphite (PG, gray spectrum) and diamond-like phase (blue spectrum).

The Raman spectrum of the laser-created structure transferred to a Cu grating (Fig. 1e) shows an expressively pronounced G-band at 1580 cm^−1^. While this band nearly dominates the spectrum, less intense vibrational resonances similar to the ones for the laser modified surface are also present. The dominance of the G-band closely resembles the Raman spectrum of HOPG^26^, therefore, indicating that the laser shockwaves induced reconstruction of the disordered arrangement of the pristine graphite into a more ordered graphitic phase. However, Selected Area of Diffraction (SAD) analysis of crystallites at multiple regions of this laser created structure (Fig. 1f) turned out being rather different from graphite, as can be noted in Fig. 2a (laser created particle) and 2**b** (polycrystalline graphite precursor). By assessing the related diffraction peaks plotted as a function of the inverse of the d-spacing (Fig. 2d), the laser induced changes are corroborated by the clear absence of the 0.338 nm interlayer distance – a signature of graphitic phase – in the created structure. Moreover, the electron diffraction pattern of this laser synthesized carbon form reasonably matches that of zinc blend diamond phase^10^,^30^ and, accordingly, lead us to argue that the laser excitation synthesized a diamond-like phase. Figure 2c exhibits a High Resolution Image corresponding to a small region of the diffraction pattern area shown in the Fig. 2a. The whole image area (16 × 16 nm^2^) of the Fig. 2c presents planes with 0.205 nm distance (diamond characteristic plane distance) and an amorphous background, which is also noticed in Fig. 2a.

**Figure 3:**
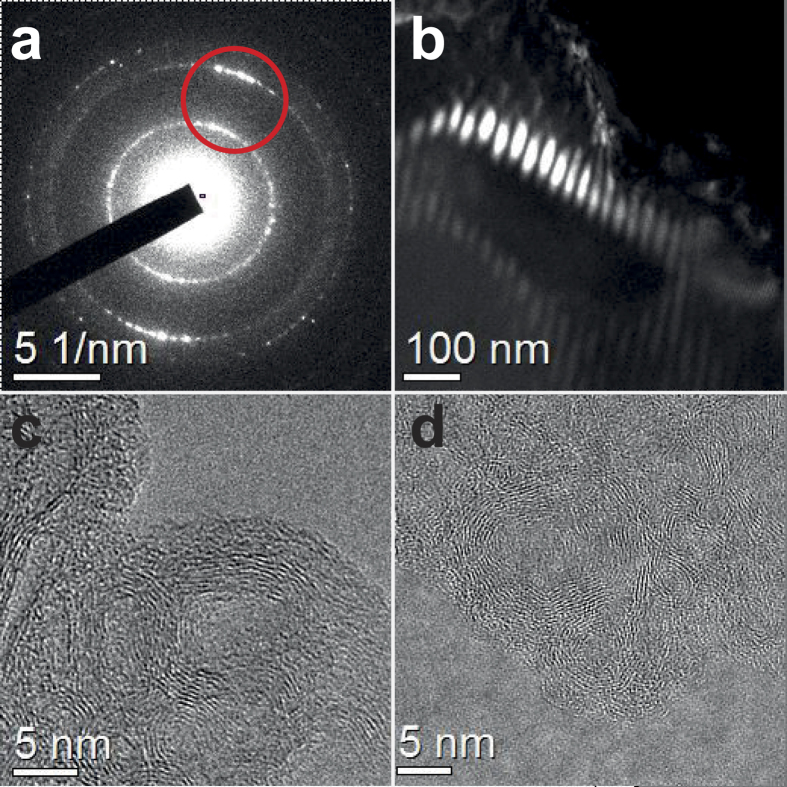
Texturized domains of the diamond-like phase and onions-like carbon coexisting at the laser created particle. (**a**) SAD of the diamond-like phase from a small area on the region of the laser created structure displayed in (**b**). The highlighted red spot in the frame (**a**) represent the selected diffraction peaks for construction the patterned domains shown in (**b**). Onion-like phases also found at the laser created structure are presented in the frames (**c**) and (**d**).

The dark field image shown in Fig. 3b was obtained using an objective aperture to select the diffraction hales corresponding to the two more intense peaks of the diamond-like phase electron diffraction pattern (Fig. 3a). Texturized micrometer domains of the latter phase (Fig. 3b) can be observed with typical sizes around 50 × 25 nm^2^. In their vicinities, onion-like structures were also encountered (Fig. 3c,d).

Here we propose that the mechanism for the transformation from graphite to the diamond-like phase follows an indirect pathway that is underpinned on the morphology of the starting material, specific thermodynamical events dictated by the fluence of overlapping ultrashort laser pulses^18^ and the formation of natural catalysts, as the onion-like structures^31^ and the laser driven nanometer sized graphite^26^. Initially, the high densities of free electrons accumulating at the boundaries of the precursor defective graphitic flakes favored the absorption of the ultrashort pulses energy steering to the creation of superexcited states, and ultimately to ablation^32^. As a consequence of the ultrafast excitation above the high fluence ablation threshold and the following explosive ablation, nonthermal shockwaves first propagate into the material after the electronic relaxation, and then heating and thermal equilibrium take place. This thermodynamical process, which happens at each laser shot, produces cumulative incremental lattice distortions^14^,^33^ leading to more ordered carbon forms^34^. Considering the exposure time with pulse superposition, these latter structures behave as transient states whose energetic barrier to phase transition lowers while the crystallinity gradually increases at each shockwave shot. Therefore, the formation of the diamond-like phase is assisted by those intermediary carbon structures which assume the role of nucleation sites more prone to phase transitions^35^.

## Conclusion

In summary, we have demonstrated, for the first time to our knowledge, the synthesis of a diamond-like phase from blasting polycrystalline graphite with spatially overlapping 25 fs ultrashort laser pulses under air atmosphere, relaxing the experimental conditions to create nanodiamonds. The diamond-like phase was found coexisting with onion-like carbon structures, in a micrometer sized translucent laser created particle that was investigated by micro Raman spectroscopy, SEM and HREM. Based on results of previous literature work and on our observations, we proposed a mechanism for the formation of the diamond-like phase from the graphite precursor. The deduced pathway of synthesis correlates the dynamics of excitation, temperature and pressure with phenomenological observations to yield relevant insights into the phase transformation of carbon allotropes by ultrafast extreme conditions.

## Methods

Samples were prepared by cutting 99.99% pure polycrystalline graphite bars (Alpha Aesar) into rectangular blocks (15 × 7 × 4 mm^3^) whose larger surfaces were manually polished with fine sandpaper up to 10 μm (RMS) roughness. Micro Raman spectroscopy (Xplora Plus microscope from Horiba with laser excitation at 532 nm), Scanning Electron Microscopy (SEM, FEI Inspect 50F), and High Resolution Electron Microscopy (HREM, JEOL JEM 2100F) were used to characterize the precursor sample and the subsequent products of the laser irradiation. To accomplish the ablation experiments, the samples were irradiated by ultrashort pulses from a Ti:Sapphire amplified laser system (Femtopower Compact Pro HR/HP from Femtolasers). This system generated 25 fs pulses centered at 780 nm, with 558 μJ of energy, in a 4 kHz pulse train. The beam was focused in air by a 75 mm focal distance achromatic doublet, and the sample was placed 1 mm before the focal plane, with the laser beam impinging on its polished face. In order to create a proper surface for the analyses, the sample was moved transversely to the beam, at 10 mm/s, in such a way to etch a line across its width; subsequently, 400 parallel lines, displaced by 10 μm, were etched and a 7 × 4 mm^2^ irradiated area was formed. At the sample surface, the calculated beam spot size (radius) was 67 μm, resulting in 3.96 J/cm^2^ fluence per pulse, which corresponds to a 1.58 × 10^14^ W/cm^2^ peak intensity. This applied fluence is three times higher than the 1.3 J/cm^2^ single shot high fluence ablation threshold that we measured for this material^36^. Moreover, we estimate that the pulses superposition were close to 300 pulses per spot, at which high fluence ablation threshold is lowered to 0.8 J/cm^2^,^36^. As a comparison, ultrashort laser pulses with fluencies as low as 60 mJ/cm^2^ are known to cause changes in the interplanar distance of the graphite^14^, a process that starts the laser ablation. After irradiation the sample was annealed (at 450 °C for 5 hours) in ambient atmosphere to eliminate debris^37^. Upon visual inspection of the ablated surface through the 10× magnification objective of the Raman microscope, the white illumination highlighted translucent micrometer-sized structures with different shapes, sizes ranging from 10 μm to 50 μm (Figures S1 to S3 of the Supplementary Information) and pronounced photoluminescence (Figure S2 of the Supplementary Information). Electrostatic attraction between those structures, which were found detached from the substrate probably due to the ablation process, and the tip of an Atomic Force Microscope, allowed their transfer to a Cu grating for the HREM analysis.

## Additional Information

**How to cite this article**: Maia, F. C. B. *et al.* Synthesis of diamond-like phase from graphite by ultrafast laser driven dynamical compression. *Sci. Rep.* **5**, 11812; doi: 10.1038/srep11812 (2015).

## Supplementary Material

Supplementary Information

## References

[b1] Baidakova M. & Vul A. New prospects and frontiers of nanodiamond clusters. J. Phys. D Appl. Phys. 40, 6300–6311 (2007).

[b2] Stoneham M. Electrons in carbon country. Nat. Mater. 3, 3–5 (2004). 14704772 10.1038/nmat1042

[b3] Lau X. C., Desai C. & Mitra S. Functionalized nanodiamond as a charge transporter in organic solar cells. Sol. Energy 91, 204–211 (2013).

[b4] Kuznetsov V. L. & Butenko Y. V. Synthesis and properties of nanostructured carbon materials: Nanodiamond, onion-like carbon and carbon nanotubes. Vol. 102 (Springer, 2003).

[b5] Shakun A., Vuorinen J., Hoikkanen M., Poikelispää M. & Das A. Hard nanodiamonds in soft rubbers: Past, present and future – A review. Compos. Part A: Appl. S. 64, 49–69 (2014).

[b6] Loir A. S. *et al.* Mechanical and tribological characterization of tetrahedral diamond-like carbon deposited by femtosecond pulsed laser deposition on pre-treated orthopaedic biomaterials. Appl. Surf. Sci. 247, 225–231 (2005).

[b7] Kaur R. & Badea I. Nanodiamonds as novel nanomaterials for biomedical applications: drug delivery and imaging systems. Int. J. Nanomed. 8, 203–220 (2013). 10.2147/IJN.S37348 PMC3544342 23326195

[b8] Maalouf R. *et al.* Characterization of different diamond-like carbon electrodes for biosensor design. Talanta 72, 310–314 (2007). 19071620 10.1016/j.talanta.2006.10.025

[b9] Erskine D. J. & Nellis W. J. Shock-induced martensitic phase transformation of oriented graphite to diamond. Nature 349, 317–319 (1991).

[b10] Hirai H. & Kondo K. Modified phases of diamond formed under shock compression and rapid quenching. Science 253, 772–774 (1991). 17835494 10.1126/science.253.5021.772

[b11] Proceedings of the National Academy of Sciences of the United States of America Kennett, D. J. *et al.* Shock-synthesized hexagonal diamonds in Younger Dryas boundary sediments. P. Natl. Acad. Sci. USA 106, 12623–12628 (2009). 10.1073/pnas.0906374106 PMC2722287 19620728

[b12] Gamaly E. G. The physics of ultra-short laser interaction with solids at non-relativistic intensities. Phys. Rep. 508, 91–243 (2011).

[b13] Jeanloz R. *et al.* Achieving high-density states through shock-wave loading of precompressed samples. P. Natl. Acad. Sci. USA 104, 9172–9177 (2007). 10.1073/pnas.0608170104 PMC1890466 17494771

[b14] Kanasaki J., Inami E., Tanimura K., Ohnishi H. & Nasu K. Formation of *sp*^3^-Bonded Carbon Nanostructures by Femtosecond Laser Excitation of Graphite. Phys. Rev. Lett. 102, 087402 (2009). 19257783 10.1103/PhysRevLett.102.087402

[b15] Hu A., Rybachuk M., Lu Q. B. & Duley W. W. Direct synthesis of sp-bonded carbon chains on graphite surface by femtosecond laser irradiation. Appl. Phys. Lett. 91, 131906 (2007).

[b16] Shirk M. D. & Molian P. A. Ultra-short pulsed laser ablation of highly oriented pyrolytic graphite. Carbon 39, 1183–1193 (2001).

[b17] Santagata A. *et al.* Carbon-Based Nanostructures Obtained in Water by Ultrashort Laser Pulses. J. Phys. Chem. C 115, 5160–5164 (2011).

[b18] Raman R. *et al.* Direct Observation of Optically Induced Transient Structures in Graphite Using Ultrafast Electron Crystallography. Phys. Rev. Lett. 101, 077401 (2008). 18764578 10.1103/PhysRevLett.101.077401

[b19] Sano T. *et al.* Femtosecond laser-driven shock synthesis of hexagonal diamond from highly oriented pyrolytic graphite. J. Phys. Conf. Ser. 165, 012019 (2009).

[b20] Nüske R. *et al.* Transforming graphite to nanoscale diamonds by a femtosecond laser pulse. Appl. Phys. Lett. 100, 043102 (2012).

[b21] Mundy C. J. *et al.* Ultrafast transformation of graphite to diamond: an ab initio study of graphite under shock compression. J. Chem. Phys. 128, 184701 (2008). 18532830 10.1063/1.2913201

[b22] Umemoto K., Wentzcovitch R. M., Saito S. & Miyake T. Body-Centered Tetragonal C_4_: A Viable *sp*^3^ Carbon Allotrope. Phys. Rev. Lett. 104, 125504 (2010). 20366546 10.1103/PhysRevLett.104.125504

[b23] Niu H. *et al.* Families of Superhard Crystalline Carbon Allotropes Constructed via Cold Compression of Graphite and Nanotubes. Phys. Rev. Lett. 108, 135501 (2012). 22540712 10.1103/PhysRevLett.108.135501

[b24] Ferrari A. C. & Robertson J. Raman spectroscopy of amorphous, nanostructured, diamond-like carbon, and nanodiamond. Philos. T. Roy. Soc. A 362, 2477–2512 (2004). 10.1098/rsta.2004.1452 15482988

[b25] Wang Y., Panzik J. E., Kiefer B. & Lee K. K. Crystal structure of graphite under room-temperature compression and decompression. Sci. Rep. 2, 520 (2012). 22816043 10.1038/srep00520 PMC3400081

[b26] Guillou C. L., Brunet F., Irifune T., Ohfuji H. & Rouzaud J.-N. Nanodiamond nucleation below 2273 K at 15 GPa from carbons with different structural organizations. Carbon 45, 636–648 (2007).

[b27] Ferroir T. *et al.* Carbon polymorphism in shocked meteorites: Evidence for new natural ultrahard phases. Earth Planet. Sc. Lett. 290, 150–154 (2010).

[b28] Amsler M. *et al.* Crystal Structure of Cold Compressed Graphite. Phys. Rev. Lett. 108, 065501 (2012). 22401083 10.1103/PhysRevLett.108.065501

[b29] Bai Y. *et al.* First-principles investigation in the Raman and infrared spectra of sp3 carbon allotropes. Carbon 78, 70–78 (2014).

[b30] Kumar A. *et al.* Formation of nanodiamonds at near-ambient conditions via microplasma dissociation of ethanol vapour. Nat. Commun. 4, 2618 (2013). 24141249 10.1038/ncomms3618

[b31] Álvarez-Murga M. *et al.* “Compressed Graphite” Formed During C_60_ to Diamond Transformation as Revealed by Scattering Computed Tomography. Phys. Rev. Lett. 109, 025502 (2012). 23030177 10.1103/PhysRevLett.109.025502

[b32] Gamaly E. G., Rode A. V., Luther-Davies B. & Tikhonchuk V. T. Ablation of solids by femtosecond lasers: Ablation mechanism and ablation thresholds for metals and dielectrics. Phys. Plasmas 9, 949–957 (2002).

[b33] Jeschke H. O., Garcia M. E. & Bennemann K. H. Theory for the Ultrafast Ablation of Graphite Films. Phys. Rev. Lett. 87, 015003 (2001). 11461471 10.1103/PhysRevLett.87.015003

[b34] Xiao J., Ouyang G., Liu P., Wang C. X. & Yang G. W. Reversible nanodiamond-carbon onion phase transformations. Nano Lett. 14, 3645–3652 (2014). 24823241 10.1021/nl5014234

[b35] Khaliullin R. Z., Eshet H., Kuhne T. D., Behler J. & Parrinello M. Nucleation mechanism for the direct graphite-to-diamond phase transition. Nat. Mater. 10, 693–697 (2011). 21785417 10.1038/nmat3078

[b36] Samad R. E., Maia F. C., Souza N. M., de Rossi W. & Vieira N. D. Determination of the Graphite Incubation Parameter in the Ultrafast Regime using the D-Scan Technique. Paper presented at *Frontiers in Optics 2014: Light-Matter Interaction, Tucson, Arizona*. 10.1364/LS.2014.LTh4I.6 (2014, October).

[b37] Osswald S., Yushin G., Mochalin V., Kucheyev S. O. & Gogotsi Y. Control of sp^2^/sp^3^ carbon ratio and surface chemistry of nanodiamond powders by selective oxidation in air. J. Am. Chem. Soc. 128, 11635–11642 (2006). 16939289 10.1021/ja063303n

