# Supplementary material for: Synthesis of diamond-like phase from graphite by ultrafast laser driven dynamical compression
Source: Sci Rep. 2015 Jul 7;5:11812. doi: 10.1038/srep11812 (PMC4493556; doi:10.1038/srep11812)
Supplement: Supplementary Information [file srep11812-s1.pdf]

Supplementary Information

for

"Synthesis of diamond-like phase from graphite by ultrafast laser driven dynamical compression"

F. C. B. Maia[1,a], R. E. Samad[2], J. Bettini[3], R. O. Freitas[1], N. D. Vieira Junior[2], N. M. Souza-Neto[1,b]

[1]Laboratório Nacional de Luz Síncrotron (LNLS), Campinas, São Paulo 13083-970, Brazil
[2]Instituto de Pesquisas Energéticas e Nucleares (IPEN-CNEN/SP), São Paulo 05508-000, Brazil
[3]Laboratório Nacional de Nanotecnologia (LNNano), Campinas, São Paulo 13083-970, Brazil
Corresponding authors:
a. francisco.maia@lnls.br
b. narcizo.souza@lnls.br

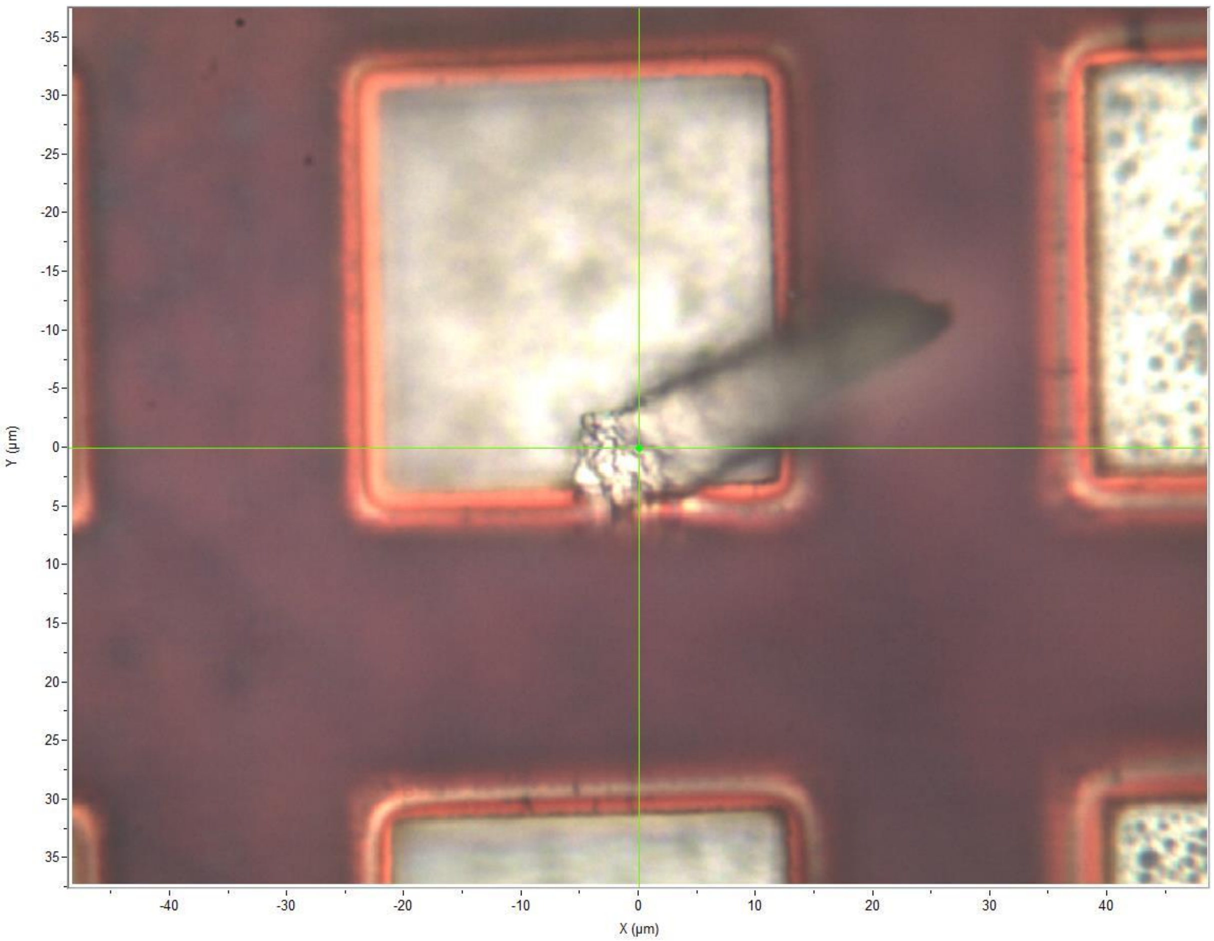

Figure S 1 – The laser created structure deposited on a Cu grating for observation on the HREM.

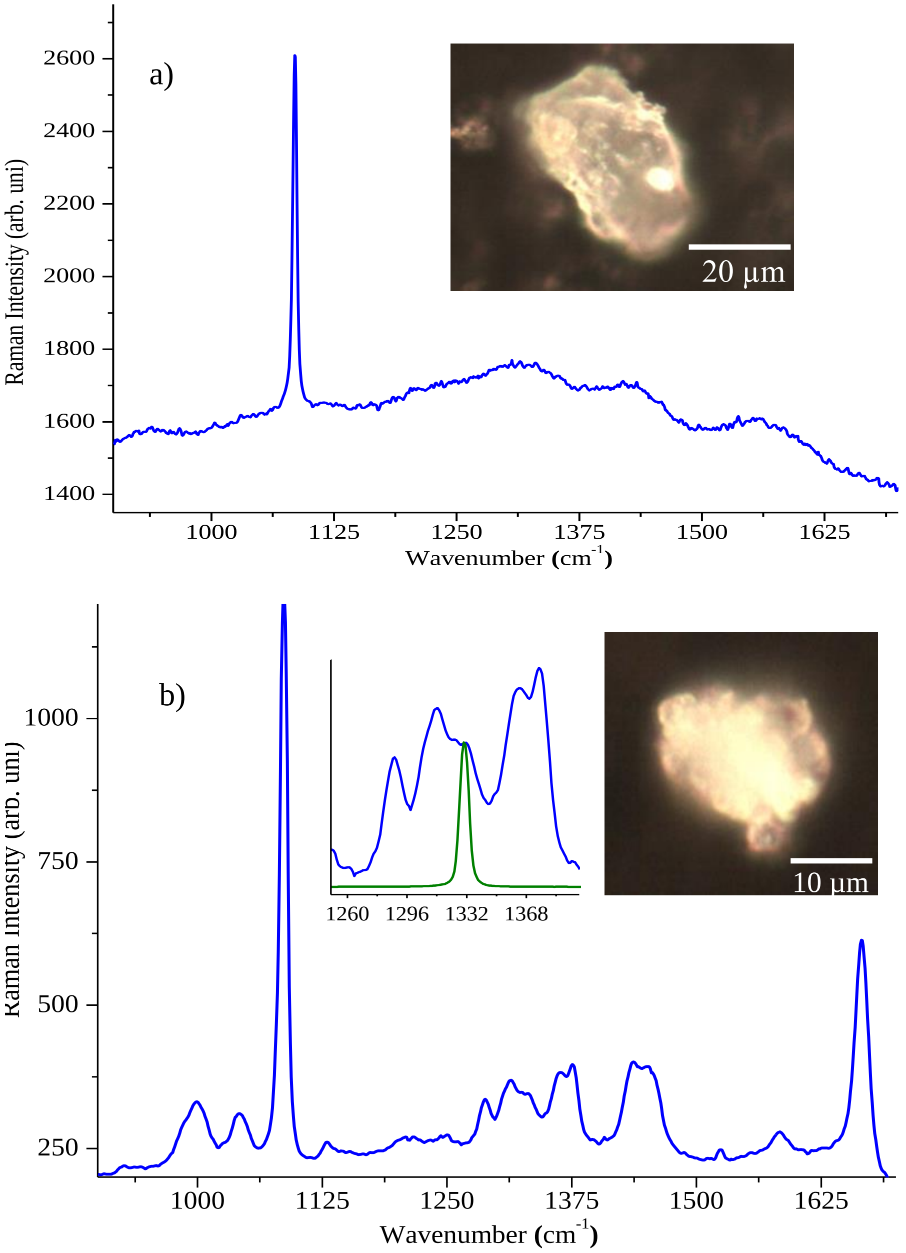

Figure S 2 –Raman spectra of two laser-created structures (the respective micrographs are shown in the insets). In a) a broadened spectral structure can be observed with a sharp prominent peak at 1085 cm^{-1} that can be attributed to the Z-carbon phase[1]. A complex spectrum of the laser particle in b) has been obtained with an expressive Z-carbon peak shifted to 1087 cm^{-1}. In the inset, a comparison between the Raman spectra of a diamond (green) and the latter laser created structure is displayed.

Figure S 2 b shows a Raman spectrum of a laser created structure, optically imaged in the respective inset, with pronounced Raman peaks centered at 990, 1000, 1130, 1042 and 1087 cm^{-1}. Such vibrational modes are absent from the known Raman resonances of pristine graphite (Figure 1a of the paper), and from cubic diamond (green curve in the inset spectrum of the Figure S 2 b). C. L. Guillou *et. al.*[2] have also reported analogous results for the recovered material from Carbon Black subjected to 15 GPa and 1900 ºC for 15 min. Complex Raman spectra were measured from shocked-meteorites systems and attributed to a new carbon-phase[3]. In these reports, the new Raman peaks were declared unexplained modes[2] and unknown vibrations concerning well-known carbon allotropes[3]. We, however, interpret our measurement as the Raman activities of laser-synthesized carbon allotropes. The 1087 cm^{-1} mode (Figure S 2 b), which is shifted to 1085 cm^{-1} in Figure S 2 a), has been attributed to the Z-Carbon phase[1] identified in hydrostatically compressed HOPG. Peaks at 1584 $cm^{-1}$and 1665 cm^{-1} can be ascribed to the G-band of the post-compressed graphite displaced to a higher frequency due to the laser-induced compression[4]. The small peaks centered at 1457 cm^{-1} and at 1522 cm^{-1} are spurious contributions from non-ideal optical isolation of the microscope. In the prominent band between 1270 and 1400 cm^{-1}, the components at 1288, 1307, 1315 and 1325 cm^{-1} are referred to vibrational modes of lonsdaleite[2,5]. We attribute the peak at 1330 cm^{-1} to the well-known mode of cubic diamond[3] (inset spectrum in Figure S 2 b). As Guillou *et. al.*[2] observed, peaks at 1364, 1376, 1437 and 1450 cm^{-1} clearly manifest. As the sample has been annealed, trans-polyacetylene related vibrations must be ruled out[5]. On the other hand, this sequence of modes can indicate a novel laser created carbon phase since it matches, within few cm^{-1}, the theoretical predictions for the fully sp^3 bonded W-carbon crystal structure[6].

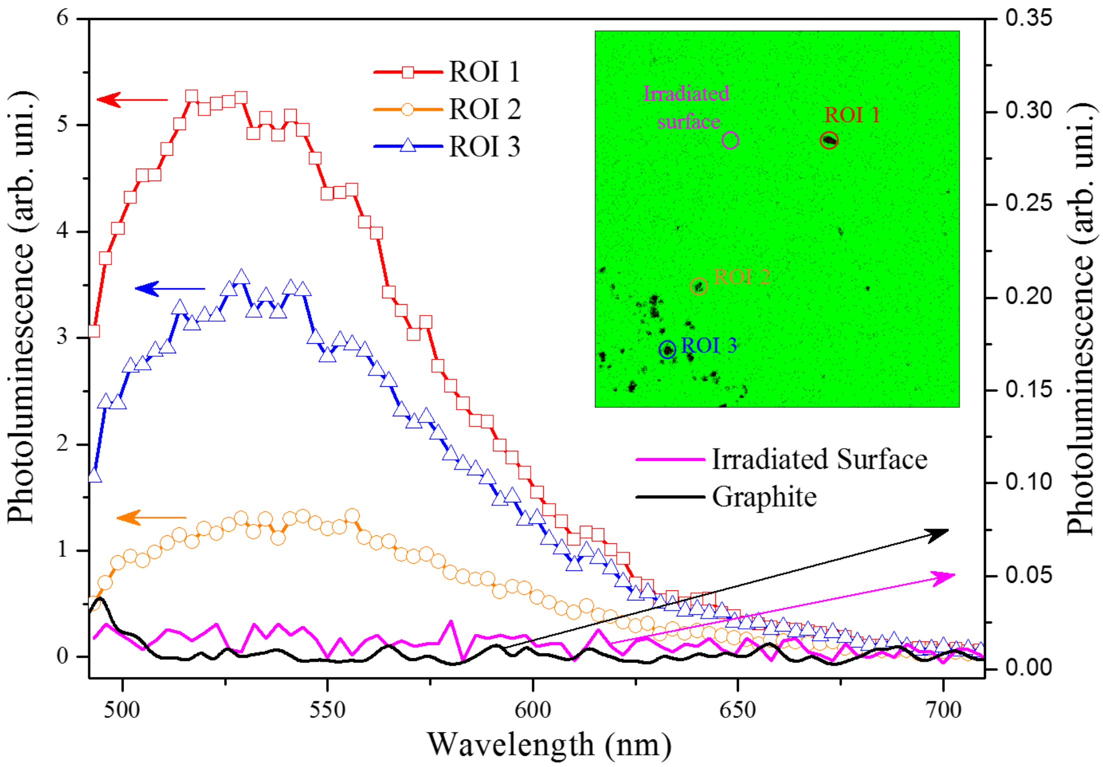

Figure S 3 –Photoluminescence spectra of three regions of interest (ROI), shown in the inset, containing laser-created structures lying on the laser modified surface. As a reference, are plotted the photoluminescences of the irradiated surface and the graphite. The inset corresponds to the spectral image at 520 nm of a 189 × 189 μm^2 sample area. These measurements were carried out by a confocal microscope (Leica TCS SP8) using a 10× objective, under laser excitation at 488 nm.

The photoluminescence spectra of the laser created particles, upon excitation at 488 nm, are neatly more intense than the laser modified surface and pristine graphite (Figure S 3). Similar photoluminescence has been reported for carbon nanoparticles[7] and nanodiamonds[8].

References

1 Amsler, M. *et al.* Crystal Structure of Cold Compressed Graphite. *Phys. Rev. Lett.* **108**, 065501 (2012).

2 Guillou, C. L., Brunet, F., Irifune, T., Ohfuji, H. & Rouzaud, J.-N. Nanodiamond nucleation below 2273K at 15GPa from carbons with different structural organizations. *Carbon* **45**, 636-648 (2007).

3 Ferroir, T. *et al.* Carbon polymorphism in shocked meteorites: Evidence for new natural ultrahard phases. *Earth Planet. Sc. Lett.* **290**, 150-154 (2010).

4 Wang, Y., Panzik, J. E., Kiefer, B. & Lee, K. K. Crystal structure of graphite under room-temperature compression and decompression. *Scientific reports* **2**, 520 (2012).

5 Goryainov, S. V. *et al.* Raman identification of lonsdaleite in Popigai impactites. *J. Raman Spectrosc.* **45**, 305-313 (2014).

6 Bai, Y. *et al.* First-principles investigation in the Raman and infrared spectra of sp3 carbon allotropes. *Carbon* **78**, 70-78 (2014).

7 Li, H. *et al.* One-step ultrasonic synthesis of water-soluble carbon nanoparticles with excellent photoluminescent properties. *Carbon* **49**, 605-609 (2011).

8 Turner, S. *et al.* Determination of Size, Morphology, and Nitrogen Impurity Location in Treated Detonation Nanodiamond by Transmission Electron Microscopy. *Adv. Funct. Mater.* **19**, 2116-2124 (2009).
